# Effect of Linear Sprints and Change-of-Direction Training Versus Small-Sided Soccer Games on Physical Performance in Highly Trained Young Female Soccer Players: A Randomized Cross-Over Study

**DOI:** 10.3390/sports13120445

**Published:** 2025-12-10

**Authors:** Abdelwahid Aboulfaraj, Fatiha Laziri, Salah Eddine Haddou, Salah Lahlou, Mohamed Aghrouch, Ali Belamjahad, Juan Del Coso, Luca Paolo Ardigò, Hassane Zouhal

**Affiliations:** 1 Natural Resources, Health and Environment Laboratory UMI, Meknès 50070, Morocco; abdelaboulfaraj@gmail.com (A.A.); f.laziri@umi.ac.ma (F.L.); 2 Regional Association of Sports Medicine Souss Massa, Agadir 80035, Morocco; salah.haddou@yahoo.com; 3 Moroccan Football Federation, Salé 11012, Morocco; s.lahlou@gmail.com; 4 Analysis Laboratory, Hassan II Regional Hospital Center, Agadir 80050, Morocco; mohamed.aghrouch@yahoo.com; 5 CETAPS UR 3832 (Centre de Recherche en Sciences du Sport), Université de Rouen Normandie, 76821 Rouen, France; belamjahadali@gmail.com; 6 Sports Science Research Centre, Rey Juan Carlos University, 28933 Madrid, Spain; juan.delcoso@urjc.es; 7 Department of Teacher Education, NLA University College, 0166 Oslo, Norway; 8 International Institute of Sport Sciences (2I2S), 35850 Irodouer, France

**Keywords:** adolescent athletes, physical conditioning, performance testing, repeated sprint ability, training adaptation, female athletes

## Abstract

Background: This study aimed to compare the effects of linear sprint training with changes of direction (LSCD) versus small-sided games (SSSG) on physical performance, agility, and soccer-specific skills in young elite female players. Methods: In a randomized crossover study, 27 players aged 15 to 17 were divided into two groups (G1 = 14, G2 = 13). After a two-week baseline period, each group completed a four-week training mesocycle (three sessions per week) consisting of either LSCD or SSG. After a two-week washout period, participants switched interventions and completed the alternate four-week mesocycle. Performance assessments were conducted before and after each mesocycle to evaluate training effects. Results: Both types of training improved physical performance, with different magnitudes. LSCD induced larger gains in sprint speed (5, 10, 20 m; *p* < 0.05), agility without the ball (*t*-test; *p* = 0.05), and explosive power (countermovement jump, repeated jumps over 15 s; *p* = 0.02 and *p* = 0.004). In contrast, SSSG led to larger improvements in aerobic endurance (Yo-Yo IR1 test; *p* = 0.03) and agility with the ball (*t*-test with ball; *p* = 0.05). No transfer effect between cycles was observed. Conclusions: In young elite female players, LSCD training was more effective in improving speed, agility, and power, while SSSG was more effective for aerobic endurance and ball agility.

**Key points:**

1. Both LSCD and SSSG training modalities improved physical performance in elite young female soccer players, though the magnitude of improvement varied depending on the specific physical capability assessed.
2. LSCD produced higher gains in sprint speed, agility and explosive power, while SSSG was more effective in enhancing aerobic endurance and ball-control agility.
3. From a practical standpoint, integrating both training approaches may offer a more comprehensive strategy for developing the diverse physical and technical demands of modern women’s soccer.

## 1. Introduction

Over the last few decades, women’s soccer has grown inexorably in popularity, with around 30 million female players worldwide [1]. During this time, the sport has experienced remarkable global development, both quantitatively, with a sharp increase in the number of licensed players [2], and qualitatively, through the professionalization of leagues and increased media coverage of major competitions such as the FIFA World Cup and the Olympic Games [3,4]. This structural change has been accompanied by an increase in the physical, technical, and tactical demands placed on top-level players, which in turn has fueled scientific interest in women’s performance in soccer [5,6]. In the field of scientific soccer research, several studies have elucidated the physical characteristics of female players in Europe and the USA [7,8]. As a result, modern women’s soccer is now recognized as a high-performance sport requiring a multi-faceted blend of physical fitness, technical skill, tactical intelligence, and psychological resilience [9,10]. Today, women’s soccer is a demanding intermittent activity involving repeated sprints, bursts, changes of direction, and other high-intensity technical actions such as jumping or kicking the ball over a match duration of around 90 min [11]. Aerobic endurance, linear sprints and changes of direction, agility related to ball handling, explosive muscular power such as vertical jumps and receptions [12], and acceleration and deceleration [13] are all fundamental physical components that significantly influence soccer performance and need to be systematically developed through targeted training interventions [14,15]. Therefore, speed development in players must target acceleration, power in straight-line running, and sprints with changes of direction in order to optimize agility and responsiveness, which are key qualities in team sports such as soccer, characterized by non-linear movements [16,17].

Small-sided soccer matches are widely used in soccer training to simultaneously develop technical, tactical, and physical skills in game-appropriate conditions [18,19]. The design of small-sided soccer games (SSSG) can vary considerably depending on several key variables, including the pitch dimensions, area per player, number of players per team and the presence or absence of goals [20,21]. Studies have shown that reducing the number of players (from 5 vs. 5 to 2 vs. 2, for example) and decreasing the size of the pitch can increase the frequency of technical actions and high-intensity efforts, while larger formats (7 vs. 7 or 8 vs. 8, for example) can better reproduce the aerobic demands of a match [22,23]. The area per player is a key determinant of physiological load, with smaller surfaces leading to higher anaerobic responses and larger surfaces favoring more sustained aerobic activity [24]. The inclusion of goals and goalkeepers has also been shown to influence player motivation, tactical behavior, and external load [25]. Despite the large body of research on SSSG, the majority of studies have been conducted on male populations, particularly professional or high-level players.

Structured training interventions targeting linear sprinting, change-of-direction speed, and agility are commonly employed in soccer to improve key physical attributes associated with match performance [26,27]. These methods often include repeated sprint training, high-intensity interval training, and changes of direction (COD) drills, which are designed to improve acceleration, deceleration, and rapid directional transitions under different loads and movement patterns. Agility-focused exercises can incorporate reactive components, such as decision-making or interaction with the ball, to better simulate game scenarios. There is evidence that these modalities can significantly improve neuromuscular coordination, explosive strength, and movement efficiency, particularly when integrated into periodized training programs [28,29]. The effectiveness of these interventions is influenced by factors such as the exercise complexity, rest intervals, surface type, and the inclusion of sport-specific stimuli. Several studies have compared the effectiveness of different training programs on the physical performance of young female soccer players, particularly the combined effects of linear sprints and changes of direction [30,31,32]. Other studies have evaluated the impacts of small-sided soccer games (SSSG) and HIIT on the physical condition of U19 players [33], as well as the benefits of plyometric training [34]. To date, the literature remains limited with regard to female soccer players aged 15 to 17. Most studies focus on U-19 players or young adults, with few direct comparisons between small-sided games (SSG) and specific programs combining linear speed and changes of direction (COD). Although some research examines SSG compared to other modalities such as HIIT, plyometrics, or endurance, it does not specifically target this age group or incorporate a structured protocol combining sprinting and CODs. This gap fully justifies exploring the comparative impact of these approaches on young female players.

Evidence of the effects of SSSG training in female athletes, particularly young or developing athletes, remains limited, highlighting a significant gap in the literature that warrants further investigation. Furthermore, there is currently insufficient evidence to determine whether structured sprint and agility training offers superior benefits, particularly in female and youth soccer players, which also highlights a significant gap in the comparative research [35].

Therefore, this randomized crossover study aims to compare the effect of two training methods—linear sprint and change of direction training (LSCD) and small-sided games (SSSG)—over an 8-week period, assessing their impact on physical performance (vertical jumps, speed, endurance, sprint repetitions), agility, and certain technical soccer skills (Loughborough Soccer Passing Test (LSPT)) in young elite female soccer players. This work addresses a gap in the current literature, which contains few comparative studies between the effects of training focused on linear speed and change of direction (COD) and those of small-sided games (SSG) in this specific population. Most existing studies have focused on participants aged 19 to 20, while research on adolescents aged 15 to 17 is still limited, even though this period represents a key phase of motor, physiological, and psychological development [36,37]. The approach adopted, combining several training modalities within a randomized crossover protocol, allows for a rigorous evaluation of their respective effectiveness, thus making an original contribution to research on performance optimization in young female soccer players.

## 2. Materials and Methods

### 2.1. Participants

The minimum sample size was calculated a priori using a statistical tool (G∗Power, version 3.1, University of Düsseldorf, Düsseldorf, Germany). The required sample size was calculated for one of our primary outcomes (i.e., 10 m sprint performance), with the following input variables: 1-β of 0.90 (power), alpha level of 0.01, and large effect size (Cohen’s f = 0.499; Cohen’s d = 0.980), based on data from a previous study [38]. The results of this analysis indicated that a total sample of *n* = 20 players would be required to perform group time interactions. The sample size was increased by 30% to account for potential dropouts during the study. Twenty-seven highly trained female soccer players from the same professional team (Hassania Union Sportif, Agadir, Morocco), playing in the Moroccan Botola 1 league, participated in the study. All the players were aged between 15 and 17 yrs. After initial assessments, participants were randomly assigned to one of two types of training, and they were divided into two groups: G1 (*n* = 14; mean age 15.4 ± 0.4 years, body mass 49.2 ± 9.7 kg, height 159.4 ± 8.5 cm, body mass index (BMI) 22.2 ± 9.7 kg/m^2^) and G2 (*n* = 13; mean age 16.7 ± 0.2 years, body mass 50.9 ± 6.5 kg, height 159.1 ± 4.7 cm, BMI 22.2 ± 5.6 kg/m^2^). All participants and their parents were informed of the potential benefits and risks before signing the consent form. The study was conducted in accordance with the latest version of the Declaration of Helsinki and was approved by the local ethics committee of Mohamed VI University in Rabat, Morocco (No. 0375/2023, 14 February 2023).

### 2.2. Experimental Design

**Figure 1:**
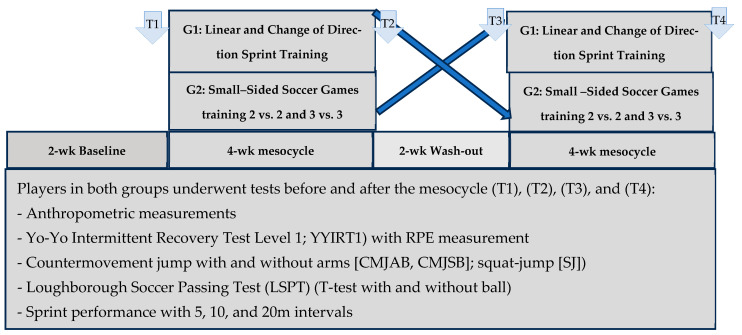
The overall study design, including different mesocycles of training and variables assessed during the 12-week cross-over experiment.

Figure 1 illustrates the general design of the randomized crossover experiment and all variables assessed during training and field testing. The experiment was conducted in the middle of the competitive season, beginning approximately eight weeks after the start of the competition phase and lasting approximately twelve weeks in total. The players were monitored and debriefed during a two-week baseline period, then randomly assigned to two experimental groups: G1 and G2. After this two-week reference period, each group completed a four-week training mesocycle (three sessions per week) consisting of either LSCD or SSG. After a two-week washout period, participants switched interventions and completed the alternate four-week mesocycle. The session rating of perceived exertion (sRPE) was assessed at the end of each session to compare the load induced by each training program. The overall training loads were equivalent for both groups for both programs. The 2-week washout period between the training programs was conducted with little training (3 sessions per week) and training sessions at 50–60% of maximum heart rate [39,40], coinciding with a break during the season. Both groups were assessed before and after each of the two experimental cross-training mesocycles, for a total of four measurement points. All tests were performed on a third-generation synthetic field, after a standardized warm-up and under the supervision of the same evaluators. All players were already familiar with the physical test protocols, as these were part of their usual training and preparation. In addition to the specific experimental training sessions, participants continued to take part in their team’s usual activities, including technical and tactical training sessions and one official match per week. However, these additional sessions were mainly intended for preparation and recovery before matches, with no specific goal of improving physical performance, which was the exclusive objective of the experimental training program.

Physical tests were conducted over three consecutive days (Table 1), with two sessions per day, to measure variables relevant to soccer performance [41,42]. Testing was conducted over a weekend and the following Monday, with two sessions per day (morning and afternoon), and all sessions were preceded by a standardized warm-up. On Saturday, anthropometric measurements were performed at 09:00, followed by the *t*-test (with and without a ball) at 11:00 to evaluate change-of-direction speed and agility. In the afternoon, the Yo-Yo Intermittent Recovery Test Level 1 (YYIRT1) was conducted at 18:00 to assess aerobic endurance. On Sunday, the morning session at 10:00 included a battery of jump tests, countermovement jumps with and without arm swing (CMJAB, CMJSB), squat jump (SJ), and 15 s repeated jumps, all of which evaluated lower-limb explosive power. The afternoon session starting at 18:00 involved linear sprint tests over 5, 10, and 20 m. On Monday, the Loughborough Soccer Passing Test (LSPT) was administered at 12:00 to assess the technical passing ability under time and accuracy constraints. The final session at 18:00 included the Repeated Shuttle Sprint Ability Test (RSSA) to evaluate anaerobic capacity and sprint repeatability [43]. Environmental conditions (temperature, surface, and equipment) were kept consistent across all sessions to ensure standardization.

### 2.3. Anthropometric Measurements

All participants underwent anthropometric assessments before and after each of the two 4-week training mesocycles (Table 2). Height was measured in centimeters (cm) using a 1 mm precision stadiometer (Seca 213^®^, Matsport, Lyon, France), body mass in kilograms (kg) using a 0.1 kg precision electronic scale (Tanita SC330 M, Matsport, Lyon, France, Japan^®^), and body fat percentage using the skinfold method. Skinfolds were measured according to the ISAK protocol by a sports nutritionist, following the protocols described by the International Biological Program [44]. Skinfold measurements were taken on six body parts: tricipital, bicipital, subscapular, suprailiac, thigh, and calf (S6SC). The body mass index (BMI) was calculated as the body mass (kg) divided by height in meters squared (kg/m^2^).

### 2.4. Fitness Variables

#### 2.4.1. Change of Direction Speed (COD) Test

The “*t*-test” is a COD test that assesses players’ ability to change direction quickly. The test was carried out according to the protocol described by Semenick [45]. The test comprises various COD movements starting with a forward sprint from point A to point B (9.14 m), a leftward run from point B to point C (4.57 m), a rightward run from point C to point D (9.14 m), a leftward run from point D to point B (4.57 m), and a backward run to the starting point from point B to point A (9.14 m) with and without a ball. The four points (A, B, C, and D) form a large “T”. Players start the test with both feet behind starting point A and complete the circuit as quickly as possible. The test was performed twice: once without the ball, to evaluate the pure change-of-direction speed, and once while dribbling a ball, to reproduce a more ecological and sport-specific situation representative of football play. The test time was recorded using the same photocell system used during the linear sprint speed test. Players completed three trials, the fastest of which was used for further analysis. The ICC for the test–retest trial for sprinting with the ball and sprinting without the ball was 0.886 and 0.906, respectively [46].

#### 2.4.2. Yo-Yo Intermittent Recovery Test Level 1 (YYIRTL1)

The test (YYIRTL1) assesses players’ endurance performance and their ability to repeat high-intensity efforts. The test consists of 2 runs of 20 m (out and back) at progressively increasing speed, separated by a 10 s recovery period controlled by a sound signal from a tape recorder. The test stopped when the players failed to reach the line before the sound signal on two consecutive occasions. The distance covered (m) was recorded as the test result. The test was preceded by a standardized 10 min warm-up on a third-generation synthetic pitch [47,48]. The ICC for test–retest reliability was 0.988 for distance covered [49].

#### 2.4.3. Jumping Performance

To measure muscle power in the lower limbs, players performed the SJ, CMJAB, and CMJSB tests and repeated 15 s jumps with arms. For the SJ test, players had to stand in a semi-crouched position for 1 s with a knee flexion angle of 90° before executing a maximal vertical jump. For the CMJ test with and without arms, players started from a standing position and had to perform a downward movement to a semi-crouched position, followed by full extension with maximum vertical acceleration. For the repeated arm jumps test, players were asked to perform free jumps for a duration of 15 s to measure the power and average jump height. For each jump test, players performed three trials separated by a 1 min interval [50]. The best of the three trials was retained for further analysis, and participants always performed their jumps in the same order (SJ, CMJ, followed by repeated jumps). The jump height was assessed using an ergonomic jump (Optojump Microgate, Guide Version 1.6.10, Software Version 1.12.23). The intra-class correlation coefficients (ICC) for the test–retest reliability were 0.936 (0.832 to 0.973) for the SJ and 0.958 (0.926 to 0.976) for the CMJ [51].

#### 2.4.4. Linear Speed Test

The players’ speeds were assessed at 5 m, 10 m, and 20 m from a standing position with a standing start. Players were asked to perform 3 maximal sprints over a distance of 20 m with a one-minute pause between trials [52]. Intermediate times at 5 m and 10 m were recorded using four photoelectric barriers (Witty Manager software version 1.4.82), placed at three distances from the starting line. The fastest time for each distance was used for further analysis. Participants were encouraged to run as fast as possible. The ICC for the test–retest reliability for performance at 5 m, 10 m, and 30 m was 0.842, 0.895, and 0.914, respectively [53].

#### 2.4.5. Loughborough Soccer Passing Test

This test was designed to assess the specific technical quality of female players according to the protocol described by Ali and colleagues [54]. Players were asked to complete a series of sixteen passes against four benches arranged in a rectangle, with a colored area (red, blue, green, and white) measuring 60 × 30 cm attached to the center of each bench, serving as the target area. Players had to make random passes against the bench of the color announced by the evaluator. Players had to complete the sixteen passes as quickly as possible, making as few mistakes as possible. The series of passes was determined by one of eight test commands randomly generated by the evaluator, so that each series of passes comprised eight short passes (3.5 m; white and red) and eight long passes (4 m; green and blue). The LSPT performance includes the result of the time taken to complete the 16 passes (initial time), the time added for errors and inaccurate passes (penalty time), and the execution time (initial time + penalty time). The penalty time is estimated according to the type of error: missed bench or wrong-colored pass (+5 s)/pass outside the target zone or handled by hand (+3 s)/pass made outside the target zone, outside the central passing zone or touching a cone (+2 s)/bonus for each second over the authorized time of 43 s (+1)/bonus for each accurate pass touching the 10 cm central strip in the middle of the target zone (−1). The time was measured using a manual stopwatch. Players completed two trials, and the best time was used as the performance score. The ICC for the test–retest reliability for the total time, penalty time, and execution time was 0.843, 9.896, and 0.921, respectively.

#### 2.4.6. Repeated Shuttle Sprint Ability Test (RSSA)

The Repeated Shuttle Sprint Ability Test (RSSA) is a crucial physical factor, as the ability to maintain intense effort over a prolonged period is frequently required at different times during a match. It is also linked to the overall performance during the match. To assess players’ ability to repeat high-intensity efforts, we carried out the RSSA. The test consisted of 6 sprints at maximum speed over a 40 m distance with a change of direction (20 m outward and 20 m return), interspersed with a 20 s recovery. Sprint times were recorded using a camera and a photocell system (Witty Manager software version 1.4.82). The best time was retained for further analysis. The percentage decrease in performance (RSSA decrement) was calculated according to the following equation: RSSA decrement = ([average sprint time]/[best sprint time] × 100) − 100) [55]. The ICC for the test–retest reliability was 0.912 for the best sprint and 0.931 for the RSSA decrement [56].

#### 2.4.7. Quantifying Training Load

The internal training load was estimated using a modified version of Borg’s rating of perceived exertion (session-RPE, sRPE) on a scale of 0–10 for the entire training session. Players were asked to provide their feelings within 15 to 20 min of the end of each training session. The sRPE was determined by multiplying the RPE index for the training session by the duration of the session. The equation load = sRPE × session volume is expressed in arbitrary units (AU) [57].

#### 2.4.8. Estimation of Menstrual Cycle Phases

As we were unable to measure estrogen (E2: 17-estradiol) and progesterone (P4) hormone concentrations [58], the players’ menstrual cycle was determined using the calendar calculation method [59]. In order to control for the potential influence of hormonal fluctuations linked to the menstrual cycle, participants were asked to declare the date of the first day of their last menstrual period and the average length of their cycle. This information was collected using a structured questionnaire, which also included questions on cycle regularity and the usual length of menstruation. Players were assumed to have a regular ovulatory menstrual cycle if the length of each cycle did not exceed a standard deviation of 3 days [58]. Using this method, it was possible to estimate whether each player was in the early follicular (EF), late follicular (LF), or mid-luteal (ML) phase at the time of testing. This estimation was performed for each of the four measurement sessions conducted in both intervention conditions. This procedure aimed to improve the reliability and reproducibility of the results by taking into account the physiological variability associated with the menstrual cycle.

#### 2.4.9. Training Program

The enrolled participants exercised for nine months of the year, with five weekly sessions of 90 min each, and a match on the weekend. During the experiment period lasting 3 months (January, February and March), three training sessions per week were dedicated to either LSCD or SSSG, and the other two sessions were dedicated to technical and tactical training. Before the players took part in the training sessions (LSCD or SSSG), a standardized warm-up was proposed (10 min general warm-up, running, arm/trunk/joint mobility/lower limb movement), 5 min dynamic stretching of the quadriceps, hamstrings, adductors, glutes, and iliopsoas, and 5 min speed-oriented exercises in a straight line and changing direction. For the two 4-week mesocycles, there were three specific training sessions per week (intervention). Training sessions took place in the afternoon at 5 p.m. after school, with a volume ranging from 60 to 80 min. All training sessions were supervised by a professional fitness trainer. Each player’s internal training load was adjusted according to the RPE, using the perceived exertion rate per session (Session-RPE) method, in accordance with the procedures defined by Impellizzeri et al. [60], and expressed in arbitrary units (AU), with two weeks’ rest between the two mesocycles and with a competitive match at the end of each training week. The 14 G1 players trained with physical exercises (LSCD) linear and/or with changes of direction with angles of 45°, 90°, and 180° with a number of repetitions and sets, recovery times, and nature (Table 3), while the G2 players trained with exercises (SSSG), (2 vs. 2 and 3 vs. 3) (Table 4).

In accordance with exercise intensities and suggested strategies [61,62], ball contact was limited to three touches in the 2 vs. 2 condition and two touches in the 3 vs. 3 condition. No goalkeeper was included, in order to enhance player involvement and increase the frequency of play [63]. The duration and format of the exercises in the SSSG condition were established to achieve an attack volume comparable to that of the LSCD condition. The exercises were designed to induce progressive variations in distance and direction throughout the training period. They were performed on reduced playing areas of 10 × 15 m, 15 × 20 m, and 20 × 30 m, respectively. During the remainder of the soccer training session, all players participated in the same activities aimed at improving individual and collective skills, in line with the envisaged randomized controlled design.

#### 2.4.10. Active Recovery Wash-Out Period

The two-week recovery program between the two mesocycles of training was in the form of an endurance game (Table 5).

### 2.5. Statistical Analysis

The data were analyzed using linear mixed-effects models to assess the effects of two interventions (linear and change-of-direction sprint training versus reduced-field soccer matches) on anthropometric and fitness outcomes. All models included a randomized intercept for participants to account for the individual variability in baseline measures, enabling more accurate and reliable estimation of the intervention effects. Models for each outcome variable included fixed effects of intervention (LSCD vs. SSSG program), time ((T1) before the first two training mesocycles, (T2) at the end of the first two training mesocycles, (T3) at the end of the two-week washout period and before crossing the two training mesocycles, (T4) at the end of the two cross-training mesocycles), and their interaction term, with time treated as a categorical variable. A random group intercept was included to account for potential baseline differences between groups. In addition, to assess the potential carry-over effects, we calculated the changes during the second intervention period (T4-T3) and compared these changes between the two groups using a linear model. Lastly, all four measurement time points (T1–T4) were included in the linear mixed-effects model. Although analyses between T2 and T3 were conducted, no significant differences were observed, confirming the effectiveness of the washout period. For clarity, these results are not presented in detail in the Results section.

The mixed-effects models were fitted using restricted maximum likelihood (REML) estimation, which optimizes the model variables while taking into account the complexity of the random effects structure. REML estimation is considered particularly suitable in the presence of random effects, as it produces unbiased estimates of variance components, even with a limited sample size. For all variables, the models tested the main effects of intervention, time, and their interaction. In each case, *p*-values were derived using Satterthwaite’s method for approximating degrees of freedom, and Cohen’s effect sizes were calculated to assess the magnitude of differences between groups and interventions with demarcations of trivial (<0.2), small (0.2–0.59), medium (0.60–1.19), large (1.2–1.99) and very large (≥2.0) [64]. Model diagnostics were performed to assess the model adequacy, including residual plots to assess normality and homoscedasticity assumptions. Significant interaction effects were further explored with pairwise comparisons. The test–retest reliability of the variables was assessed using Cronbach’s model of ICCs and SEMs according to the method of Peltola [65]. All statistical analyses were performed using R software (R Core Team (2024). R: a language and environment for statistical computing [Computer software], extracted from (https://cran.r-project.org/, accessed on 13 April 2024)) with a significance level set at *p* < 0.05.

## 3. Results

### 3.1. Descriptive and Baseline Comparisons

The baseline comparisons of anthropometric variables revealed no significant differences between the groups at the pre-intervention measurements (all *p* > 0.05). For fitness variables, most were not significantly different, with the exception of S10 (*p* = 0.02), CMJ without arm (*p* = 0.02) and *t*-test without ball (*p* = 0.002). With regard to the significant baseline differences identified in three fitness variables, these were appropriately addressed in the modeling used in the present study, where the linear mixed model accounted for individual variations between groups. In addition, carry-over effects were modeled to ensure that any influence of interventions in the cross-over design was properly managed, minimizing any bias in the interpretation of results.

### 3.2. Anthropometric Characteristics

Overall, the analysis of the height, body mass, BMI, and S6SC revealed no significant differences between interventions or between time points (Table 6).

Data are presented as the mean and standard deviation. BMI: body mass index, S6SF: 6 Skinfold measurements, LSCD: linear sprint and change of direction, SSSG: small-sided soccer games. For G1 and G2, comparisons between LSCD and SSSG showed no significant differences in any of the anthropometric variables (height, body mass, BMI, and S6SC). Specifically, the *p*-values were all higher than 0.05 (e.g., body height: *p* = 0.94, body mass: *p* = 0.94, BMI: *p* = 0.94, and S6SC: *p* = 0.84 for G1; body height: *p* = 0.92, body mass: *p* = 0.96, BMI: *p* = 0.96, and S6SC: *p* = 0.94 for G2). Cohen’s d values indicated negligible effects for all variables, suggesting minimal differences between interventions in either group. No carry-over effects were detected for any of the anthropometric variables. The interaction terms between the intervention and time were all non-significant, suggesting that the changes observed during the second intervention phase were not influenced by the effects of the first intervention. This result confirms the idea that the elimination period avoids carry-over effects for anthropometric measurements.

### 3.3. Fitness Variables

**Figure 2:**
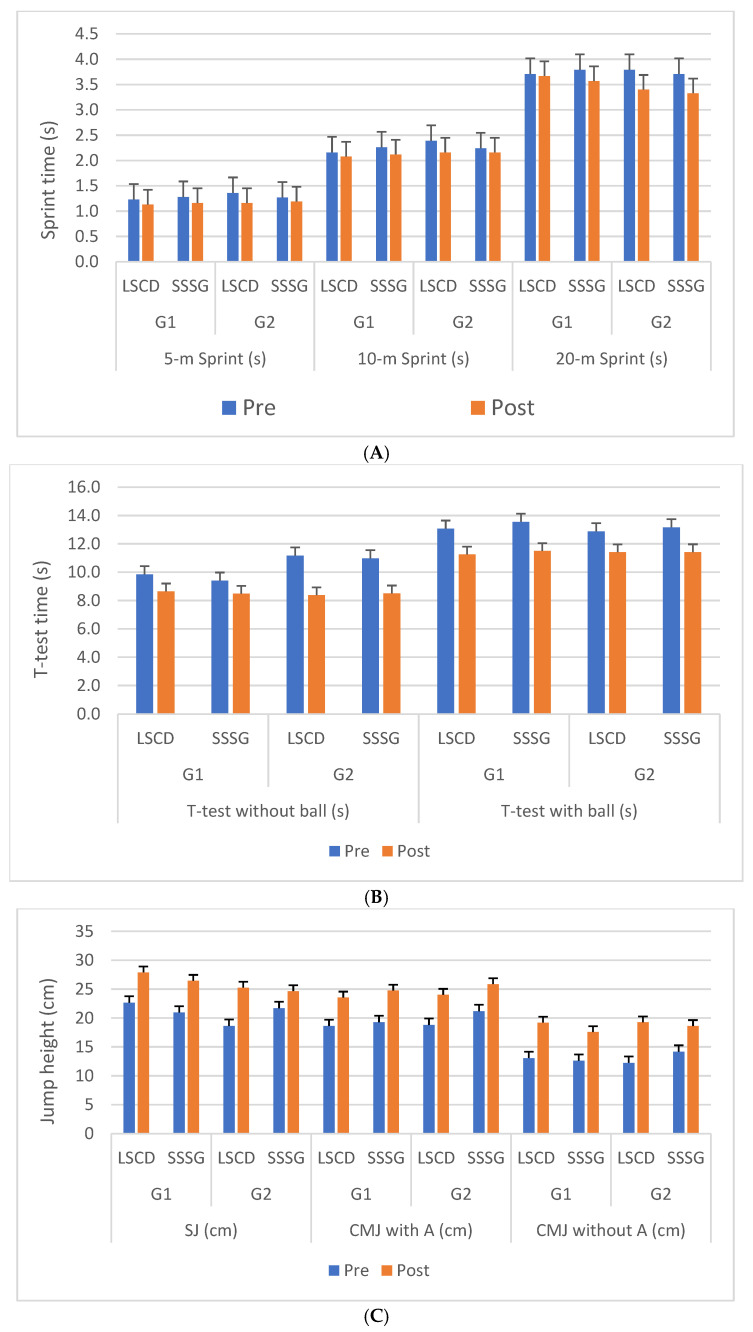
Effects of LSCD training compared to SSG training over 8 weeks on fitness variables. (**A**) Sprint time (s) for distances of 5 m, 10 m, and 20 m. (**B**) Performance time in the agility *T* test (s), with and without a ball. (**C**) Vertical jump height (cm) in the squat jump (SJ), countermovement jump with arms (CMJ with A) and countermovement jump without arms (CMJ without A). Data are presented as the mean and standard deviation. SJ: squat jump, CMJ with A: countermovement jump with arm, CMJ without A: countermovement jump without arm, LSCD: linear sprint and change of direction, SSSG: small-sided soccer games.

When comparing the effects of the two interventions, LSCD and SSSG, distinct response patterns were observed between the two groups (Table 7 and Figure 2). Group 1 generally showed larger improvements than Group 2 across several fitness outcomes, particularly in sprint performance (5 m, 10 m), agility, and reactive strength. Significant differences between-intervention were observed in various sprint and jump measures (*p* < 0.05) with small-to-large effect sizes, especially in G1.

G1 showed significant improvements in the 5 m and 10 m sprint times following both interventions, with larger within-group effects after SSSG (*d* = −2.4 for 5 m, −2.33 for 10 m) than LSCD (*d* = −2.0 and −1.33, respectively). However, the between-intervention effect sizes slightly favored SSSG for the 10 m sprint in Group 1 (*p* = 0.002, *d* = −1.33). In contrast, Group 2 improved more after LSCD, particularly in the 5 m and 10 m sprints (*d* = −2.22 and −2.3, respectively), while SSSG yielded smaller improvements. For the 20 m sprint, both groups improved significantly after both interventions (*p* < 0.001), but between-intervention differences were not significant, suggesting a similar efficacy of LSCD and SSSG over longer sprint distances.

Significant improvements were observed in the *t*-test performance (with and without the ball) for both groups following both interventions. Notably, LSCD produced slightly larger effects in Group 2 (*d* = −4.2 vs. −3.5 for SSSG, without ball). However, the intervention comparisons showed no significant differences (all *p* > 0.05), indicating comparable effectiveness.

Both interventions elicited large improvements in jump-based outcomes. In Group 1, SSSG produced slightly higher gains in squat jump (SJ) and countermovement jumps with and without arms (CMJAB and CMJSB), although LSCD was also effective. For Group 2, LSCD led to substantially larger improvements in CMJSB (*d* = 5.01 vs. 3.02 for SSSG), with a significant intervention effect (*p* = 0.04, *d* = −0.91), favoring LSCD.

The reactive jump performance consistently improved across both interventions, but Group 2 benefited more from LSCD, particularly for high-load explosive tasks. For instance, Group 1 showed stronger responses to SSSG for short-distance sprints and jumps, but LSCD also produced large effects, especially in power-based tests (e.g., CMJSB: *d* = 8.25 LSCD vs. 6.55 SSSG). Group 2, however, demonstrated a higher benefit from LSCD, especially in sprinting and reactive jump tasks. Across groups, LSCD tended to enhance linear speed and power, while SSSG was more effective for agility and endurance in Group 2. YYIRT1 (Yo-Yo Intermittent Recovery Test) improvements favored SSSG in Group 2 (*p* = 0.03, *d* = 0.56), highlighting the endurance-specific benefits of SSSG-style training. No clear superiority was observed between interventions in terms of the RSSA.

No significant carryover effects were observed for physical fitness variables, suggesting that the washout period between interventions was sufficient to minimize any residual effects from the preceding training phase. However, the observed within-group differences indicate that Group 1 responded more favorably to SSSG, while Group 2 showed larger improvements following LSCD. These patterns highlight the importance of individual and group-level variability in training adaptation, as well as the strength of the crossover design in isolating the effects of each intervention.

### 3.4. Phases of the Menstrual Cycle

The distribution of players according to the phase of the menstrual cycle was estimated based on the start date of their period during the four physical tests (T1–T4) for the LSCD and SSSG interventions (Table 8). No statistically significant differences in physical performance were observed between the phases of the menstrual cycle. It is important to note that the estimation of phases was based solely on the start date of menstruation, which is an approximate method for accurately identifying the ovulatory and luteal phases. In addition, the small size of the subgroup of players in the ovulatory phase may have limited the detection of possible variations in performance.

### 3.5. Session RPE

No significant differences in sRPE were observed between G1 and G2 across any of the four weeks in either mesocycle (Table 9 and Table 10). During mesocycle 1, sRPE values were similar between G1 and G2 in week 1 (*p* = 0.95, *d* = 0.001), week 2 (*p* = 0.93, *d* = −0.02), week 3 (*p* = 0.56, *d* = −0.05), and week 4 (*p* = 0.94, *d* = −0.03). Likewise, during mesocycle 2, no significant between-group differences were found in week 1 (*p* = 0.16, *d* = −0.04), week 2 (*p* = 0.70, *d* = 0.10), week 3 (*p* = 0.53, *d* = 0.22), or week 4 (*p* = 0.57, *d* = −0.09). Overall, sRPE responses remained statistically comparable between groups throughout the intervention, with all effect sizes classified as trivial (*d* < 0.20; Table 9 and Table 10)

## 4. Discussion

This is the first randomized crossover study to examine the effects of two training methods, linear sprint with change of direction (LSCD) and small-sided soccer games (SSSG; 2-on-2 and 3-on-3 formats), on anthropometric variables and physical performance in well-trained young Moroccan female soccer players. It is important to note that the study included two groups that underwent both training interventions (LSCD and SSSG) in a randomized crossover design. Therefore, the discussion focuses on the effects of each training program rather than the differences between the groups. The main results showed that both eight-week interventions led to improvements in physical fitness, although the magnitude and specificity of these effects varied depending on the outcome. LSCD was particularly effective in improving the sprint speed, explosive power, and agility [66], while SSSG resulted in notable gains in aerobic endurance, agility with ball control, and reactive strength. These results confirm the usefulness of LSCD and SSSG as effective training modalities in youth soccer development programs.

### 4.1. Anthropometric Characteristics

With regard to the anthropometric results and body composition, no significant differences were observed between the two training models. This is consistent with previous studies that reported no significant changes in body composition after lower-body plyometric training of similar duration [67]. These results suggest that short-term interventions may be insufficient to induce measurable anthropometric adaptations in already trained individuals.

### 4.2. Physical Fitness Variables

Comparative studies between LSCD and SSSG are limited, particularly with regard to performance outcomes. Most previous research has focused independently on linear sprints or change of direction (COD) exercises [59,62]. Our study indicates that both the LSCD and SSSG protocols significantly improved physical performance measures after the eight-week intervention, with LSCD demonstrating superior improvements in sprint- and power-related tasks.

Sprinting, whether straight or with changes of direction, is an essential component of performance in soccer. The LSCD protocol introduced a new training format combining repeated linear sprints and changes of direction. Following the LSCD intervention, the G1 group showed significant and larger improvements in sprint performance over 5, 10, and 20 m, COD, CMJ, PRJ15′, and HRJ15′. More specifically, the *t*-test without ball (*p* = 0.05, *d* = 0.46), CMJ (*p* = 0.02, *d* = 0.52), and PRJ15′’ (*p* = 0.004, *d* = 0.85) showed moderate to large effects, reflecting improvements in anaerobic power and reactive strength.

These results are consistent with those of previous studies conducted on prepubescent athletes, which demonstrated improved sprint performance over 10 to 30 m after 12 weeks of linear sprint training [61,68]. Two recent systematic reviews also reported moderate to large improvements in sprint speed, with effects ranging from 0.43 to 1.59, although the stage of maturation introduced some heterogeneity [69,70]. Other studies corroborate these findings, with significant gains in the 30 m sprint, CMJ, and jump tests observed in elite soccer players under the age of 15 after shuttle sprint training [71] and improved sprint and COD performance after six weeks of training combining COD exercises with and without a ball [72]. Similar benefits were observed in children aged 10 to 12 who underwent eight weeks of COD and plyometric training [73] and in agility and jump measurements after linear sprint training [74].

These improvements are likely due to neuromuscular and biomechanical adaptations, such as increased stride length [75], improved coordination [76], reduced ground contact time, and increased musculotendinous stiffness [77]. Increased lower limb strength and ground reaction forces have also likely contributed to these improvements [78]. The improvement in explosive power and reactive strength may be due to higher efficiency in the stretch-shortening cycle, characterized by faster transitions between eccentric and concentric muscle actions [28]. In addition, improvements in acceleration, deceleration, and COD performance are associated with neuromuscular qualities through underlying mechanisms such as neuromuscular adaptation and muscle fiber recruitment, which are induced by linear sprint training and training involving changes of direction (COD) due to the significant demand placed on fast-twitch type II muscle fibers (IIa and IIx), due to their ability to rapidly generate high force during acceleration phases. This demand promotes the recruitment of high-threshold motor units and leads to neuromuscular adaptations such as an increase in motor neuron firing frequency and improved inter- and intramuscular synchronization [79,80]. The mechanical stresses specific to sprints with COD, particularly during the eccentric deceleration phases, reinforce these adaptations by inducing selective hypertrophy of fast fibers and improving the intramuscular calcium regulation system, facilitating faster and more powerful contraction [81,82].

In contrast, SSSG training in group G2 yielded significantly better results than LSCD alone in terms of aerobic endurance (moderate effect, *d* = 0.55) and ball agility (*t*-test, *p* = 0.05). These results are consistent with previous findings showing that SSSG training improves aerobic capacity, motor coordination, and agility in conditions representative of a match [61,83].

The modest or negligible improvements observed in Group G2 in other performance domains are consistent with these findings. Several studies have reported that training through small-sided games (SSG) presents important limitations for enhancing maximal sprint speed and maximal muscular power in young female soccer players [84,85]. Maximal sprinting performance relies on high-intensity linear efforts that require substantial activation of type IIx muscle fibers, maximal horizontal force production, and specific intermuscular coordination during acceleration and maximal velocity phases [86,87]. Such neuromuscular demands are rarely replicated within SSG formats, where high-speed running bouts are typically short and interspersed with frequent changes of direction, decelerations, and close technical interactions [88]. This limited mechanical and energetic specificity of SSG compared to linear sprinting largely explains the absence of significant improvements in neuromuscular qualities observed in our study. SSGs primarily stimulate aerobic and glycolytic systems due to the repeated execution of submaximal intermittent efforts. Consequently, these games are more conducive to the development of aerobic capacity, metabolic fatigue tolerance, and reactive strength, particularly through low-amplitude plyometric actions and repeated accelerations or decelerations [89,90].

Therefore, while SSGs represent an effective and ecologically valid training method to enhance soccer-specific endurance, tactical decision-making, and reactive agility, their ability to induce maximal neuromuscular adaptations—particularly in sprint speed and muscular power—remains limited in young female players [84,87,91].

This is in line with conclusions that emphasize that sprint-specific technical training is more effective for developing speed than small-sided games [92]. Furthermore, one study highlights the importance of repeating efforts close to maximum speed, with high intensity and sufficient recovery times, conditions that are rarely met in SSGs [93]. In addition, maximum muscle power is rarely used in these small-sided games. Recent studies show that resistance sprints, particularly with sleds, lead to significant improvements in acceleration and maximum speed in female soccer players, unlike training based solely on playing the game [94]. Furthermore, accumulated fatigue and lack of recovery during SSG can reduce the effective intensity of the effort, thereby limiting neuromuscular adaptations, as highlighted in a meta-analysis [95], which concludes that HIIT training protocols are generally more effective than SSG for developing linear sprint speed.

The menstrual cycle, a fundamental biological rhythm after the circadian cycle, influences female physiology and athletic performance through fluctuations in estradiol (E2) and progesterone (P4) [96,97]. These hormonal variations, spread across the menstrual, follicular, ovulatory, and luteal phases, modulate metabolism, muscle recovery, thermoregulation, cardiovascular function, and psychological well-being [98]. They can also influence soccer performance due to their effects on energy availability, neuromuscular coordination, and exercise tolerance [9,99]. Despite an abundance of literature, the results remain mixed, with several studies showing no significant variation according to the phases of the cycle [100]. These findings highlight the need to integrate hormonal fluctuations as a central physiological factor in the analysis of female performance [101]. However, in the context of this study, the variations in performance observed between G1 and G2 after the implementation of the two programs can be attributed to the specific adaptations induced by these training methods. Thus, the improvements observed in the current study do not appear to be the result of hormonal fluctuations related to the menstrual cycle but rather the specific effect of the programmed interventions on physical qualities and soccer performance.

The scientific interpretation of the similar overall training load and sRPE between the two groups shows that both groups maintained almost identical values per session each week, as well as an equivalent overall training load. This consistency suggests that exposure to training load was strictly controlled, allowing for a reliable comparison of the effects of the interventions. The perceived training load, measured by the RPE multiplied by session duration [57], is a validated and widely used indicator in the context of soccer to estimate internal load. The homogeneity of the RPE values between groups suggests that the perception of effort was similar, allowing us to exclude the influence of perceived load as a factor of variation between groups [102]. From a methodological point of view, rigorous control of training load ensures that any differences in physiological, technical, or performance adaptations observed at the end of the intervention are likely due to the specific content of the two programs and not to a difference in perceived volume or intensity [103]. This reinforces the internal validity of the study. Finally, this observation also has practical implications: it demonstrates that it is possible to manipulate program content (type of exercises or tactical structure) without necessarily changing the perceived load, which may allow coaches to target specific adaptations while maintaining consistent management of the overall training load [104]. These limits can be influenced by tactical requirements, individual motivation, and the ability to reproduce high-intensity actions during play. It therefore appears essential to supplement this type of training with targeted exercises, including linear sprints, sprints with changes of direction, and specific muscle strength work in order to optimize neuromuscular performance in young female soccer players. Collectively, our results confirm that LSCD and SSSG both offer unique and complementary benefits. LSCD proved to be more effective for high-intensity and explosive performance measures, while SSSG improved endurance and sport-specific agility. This confirms the value of an integrated approach to physical conditioning, combining both modalities in a periodized training model to optimize the athletic development of young players [105].

## 5. Limitations

This study presents several limitations that should be acknowledged. First, the sample consisted exclusively of elite female youth soccer players with no prior experience in structured strength training. Therefore, the findings cannot be generalized to other populations, such as male or adult athletes. Second, the absence of external load quantification through GPS data for both training methods limited our ability to analyze the specific physical demands associated with each model (i.e., LSCD vs. SSSG), particularly regarding key soccer performance variables such as accelerations, decelerations, and sprinting actions. Third, several physiological parameters were not monitored during the experiment, including hormonal responses, inflammatory markers, and indicators of muscle damage, which could have provided deeper insight into the underlying adaptation mechanisms. Moreover, factors such as sleep quality and psychological stress, which are known to influence performance outcomes, were not systematically controlled. Finally, the potential combined effect of both training approaches (LSCD and SSSG) was not analyzed. It remains possible that an integrated program combining both methods could represent a more effective strategy for optimizing performance adaptations in elite youth players.

## 6. Conclusions

This randomized crossover study demonstrated that both linear sprint and change-of-direction (LSCD) training and small-sided soccer games (SSSG) are effective in improving physical performance in highly trained young female soccer players. However, the magnitude and specificity of adaptations differed between the two modalities. LSCD training produced larger improvements in sprint speed, explosive power, and agility without the ball, while SSSG training was more effective in enhancing aerobic endurance and agility with ball control. These findings suggest that each training method targets distinct performance domains, suggesting the high specificity of training and the adaptations induced. Although each method targets different performance domains, their combined use may provide a more holistic approach to player development. However, this integrative strategy was not directly tested in the present study. Therefore, future research should investigate the effects of concurrent or sequential implementation of LSCD and SSSG within a periodized training framework to determine whether their combination yields superior adaptations compared to either method alone.

## 7. Practical Applications

The results of this study offer valuable insights for applied practice in youth elite soccer. Coaches and performance staff can use both LSCD and SSSG training methods as effective in-season tools to maintain and enhance players’ physical performance without compromising recovery. LSCD sessions should be prioritized when the objective is to improve linear sprinting speed and acceleration mechanics, while SSSG sessions are particularly suitable for developing technical–tactical skills and game-related endurance within realistic contexts. Importantly, alternating these two methods or combining them within the same training mesocycle could yield complementary adaptations that better reflect the multidimensional demands of football. When implementing these strategies, practitioners should also consider individual load monitoring (e.g., sRPE) to ensure appropriate recovery, minimize fatigue, and sustain high performance throughout the competitive season.

## Figures and Tables

**Table 1:** Timetable for physical and biological tests.

	Saturday	Sunday	Monday
Morning 9:00–12:00 a.m.	Anthropometric measurements *t*-Test with and without the ball 11:00 a.m.	CMJ without arm swing CMJ with arm swing SJ 15 s repeated jumps	LSPT
Afternoon 18:00–19:00 p.m.	YYIRT1	5 m, 10 m, and 20 m sprint test	RSSA

Abbreviations: CMJ = countermovement jump; SJ = squat jump; YYIRT1 = Yo-Yo Intermittent Recovery Test Level 1; LSPT = Loughborough Soccer Passing Test; RSSA = Repeated Shuttle Sprint Ability.

**Table 2:** Characteristics of study participants (group 1, G1, and group 2, G2) measured at T1, T2, T3, and T4. Values are means and standard deviations. BMI: body mass index; S6SF: six skinfold measurements.

Group	Variable	T1	T2	T3	T4
G1	Height (cm)	159.4 ± 8.5	159.4 ± 8.5	159.8 ± 8.4	159.8 ± 8.4
	Body mass (kg)	49.2 ± 9.7	50.0 ± 9.8	50.5 ± 9.7	50.6 ± 9.6
	BMI (kg/m^2^)	22.2 ± 9.7	22.5 ± 9.8	22.6 ± 9.7	22.8 ± 9.6
	S6SF	80.9 ± 26.4	81.9 ± 26.6	82.0 ± 26.9	81.9 ± 27.0
G2	Height (cm)	159.1 ± 4.7	159.2 ± 4.7	159.3 ± 4.7	159.4 ± 4.6
	Body mass (kg)	50.9 ± 6.5	51.4 ± 6.3	51.3 ± 6.2	50.9 ± 5.7
	BMI (kg/m^2^)	22.2 ± 5.6	22.5 ± 6.1	23.4 ± 6.1	23.8 ± 6.0
	S6SF	82.2 ± 23.7	82.9 ± 23.7	83.4 ± 23.6	84.1 ± 23.7

**Table 3:** Description of the linear sprint and change of direction (LSCD) training program.

	WEEK 1	WEEK 2	WEEK 3	WEEK 4
	P1: Linear Sprints and Changes of Direction Training Program Description (LSCD)			
Session 1	Speed-oriented warm-up 15′ 2 × 30 m, Linear Sprint 2 × 30 m, with 180° COD 2 × 30 m, with 90° COD r: TW × 15 R: 3′ 12′ cooling-down	Speed-oriented warm-up 15′ 2 × 30 m, Linear Sprint 2 × 30 m, with 45° COD 2 × 30 m, with 90° COD r: TW × 15 R: 3′ 12′ cooling-down	Speed-oriented warm-up 15′ 2 × 30 m, Linear Sprint 30 m, with 180° COD 30 m, with 90° COD 30 m, with 45 °COD r: TW × 15 R: 3′ 12′ cooling-down	Speed-oriented warm-up 15′ 2 × 30 m, Linear Sprint 30 m, COD 180° 30 m, with 90° COD 30 m, with 45° COD r: TW × 15 R: 3′ 12′ cooling-down
Session 2	Speed-oriented warm-up 15′	Speed-oriented warm-up 15′	Speed-oriented warm-up 15′	Speed-oriented warm-up 15′
	2 × 20 m, Linear Sprint	2 × 20 m, Linear Sprint	2 × 20 m, Linear Sprint	2 × 20 m, Linear Sprint
	2 × 20 m, with 180° CD	2 × 20 m, with 180° CD	2 × 20 m, with 180° CD	2 × 20 m, with 180° CD
	2 × 20 m, with 90° CD	2 × 20 m, with 90° CD	2 × 20 m, with 90° CD	2 × 20 m, with 90° CD
	2 × 20 m, with 45° CD	2 × 20 m, with 45° CD	2 × 20 m, with 45° CD	2 × 20 m, with 45° CD
	r: TW × 15	r: TW × 15	r: TW × 15	r: TW × 15
	R: 3′	R: 3′	R: 3′	R: 3′
	12′ cooling-down	12′ cooling-down	12′ cooling-down	12′ cooling-down
Session 3	Speed-oriented warm-up 15′	Speed-oriented warm-up 15′	Speed-oriented warm-up 15′	Speed-oriented warm-up 15′
	3 × 10 m, Linear Sprint	3 × 10 m, Linear Sprint	3 × 10 m, Linear Sprint	3 × 10 m, Linear Sprint
	3 × 10 m, with 180° CD	3 × 10 m, with 180° CD	3 × 10 m, with 180° CD	3 × 10 m, with 180° CD
	3 × 10 m, with 90° CD	3 × 10 m, with 90° CD	3 × 10 m, with 90° CD	3 × 10 m, with 90° CD
	3 × 10 m, with 45° CD	3 × 10 m, with 45° CD	3 × 10 m, with 45° CD	3 × 10 m, with 45° CD
	r: TW × 15	r: TW × 15	r: TW × 15	r: TW × 15
	R: 3′	R: 3′	R: 3′	R: 3′
	12′ cooling-down	12′ cooling-down	12′ cooling-down	12′ cooling-down

The LSCD training program was conducted over four weeks, with three sessions per week. Each session began with a 15 min speed-oriented warm-up, followed by a combination of linear sprints and sprints incorporating changes of direction (CoD) at 45°, 90°, and 180°, performed over distances of 10 m, 20 m, and 30 m depending on the session. Exercises were completed in sets with a work-to-rest ratio of 1:15 and 3 min of recovery between sets. Each session concluded with a 12 min cool-down.

**Table 4:** Small-sided soccer games (SSSG) training program description.

	WEEK 1	WEEK 2	WEEK 3	WEEK 4
Session 1	Coordination warm-up with ball 6′	Coordination warm-up with ball 6′	Coordination warm-up with ball 6′	Coordination warm-up with ball 6′
	3 sets 3 × 3′ (3C3)	3 sets 3 × 3′ (3C3)	3 sets 3 × 3′ (3C3)	sets 3 × 3′ (3C3)
	(85–90% HR max)	(85–90% HR max)	(85–90% HR max)	(85–90% HR max)
	r: 2′–3′	r: 2′–3′	r: 2′–3′	r: 2′–3′
	R: 5′–6′	R: 5′–6′	R: 5′–6′	R: 5′–6′
	8′ cooling-down	8′ cooling-down	8′ cooling-down	8′ cooling-down
	Field size: 30 m × 20 m	Field size: 30 m × 20 m	Field size: 30 m × 20 m	Field size: 30 m × 20 m
Session 2	Coordination warm-up with ball 6′	Coordination warm-up without ball 6′	Coordination warm-up with ball 6′	Coordination warm-up without ball 6′
	3 sets 4 × 1′30″ (2C2)	3 sets 3 × 3′ (3C3)	3 sets 4 × 1′30″ (2C2)	3 sets 3 × 3′ (3C3)
	(85–90% HR max)	(85–90% HR max)	(85–90% HR max)	(85–90% HR max)
	r: 2′–3′	r: 2′–3′	r: 2′–3′	r: 2′–3′
	R: 5′–6′	R: 5′–6′	R: 5′–6′	R: 5′–6′
	8′ cooling-down	8′ cooling-down	8′ cooling-down	8′ cooling-down
	Field size: 15 m × 20 m	Field size: 30 m × 20 m	Field size: 15 m × 20 m	Field size: 30 m × 20 m
Session 3	Coordination warm-up with ball	Coordination warm-up without ball	Coordination warm-up with ball	Coordination warm-up without ball
	3 sets 4 × 1′30″ (2C2)	2 sets 4 × 4′ (2C2)	3 sets 4 × 1′30″ (2C2)	2 sets 4 × 4′ (2C2)
	(85–90% HR max)	(85–90% HR max)	(85–90% HR max)	(85–90% HR max)
	r: 2′–3′	r: 2′–3′	r: 2′–3′	r: 2′–3′
	R: 5′–6′	R: 5′–6′	R: 5′–6′	R: 5′–6′
	6′ cooling-down	6′ cooling-down	6′ cooling-down	6′ cooling-down
	Field size: 15 m × 20 m	Field size: 15 m × 20 m	Field size: 15 m × 20 m	Field size: 15 m × 20 m

The SSSG training program was conducted over four weeks with three sessions per week. Each session began with a coordination-focused warm-up (with or without the ball), followed by small-sided games in 2 vs. 2 or 3 vs. 3 formats, performed in sets of short, high-intensity bouts (85–90% HRmax). Game durations ranged from 1′30′’ to 3′ per bout, with rest intervals of 2–3 min between repetitions and 5–6 min between sets. Games were played on small fields (15 × 20 m or 30 × 20 m), and sessions concluded with an 8-min cool-down.

**Table 5:** Training program during the 2-week washout period.

	WEEK 1	WEEK 2
Session 1	8′ coordination warm-up with ball	8′ Coordination warm-up without ball
	Aerobic training 3 × 10′	Aerobic training 3 × 10′
	(85–90% HR max)	(85–90% HR max)
	R: 4′	R: 4′
	10′ cooling-down	10′ cooling-down
Session 2	8′ coordination warm-up with ball	8′ coordination warm-up without ball
	Aerobic training 3 × 7′	Aerobic training 3 × 7′
	(85–90% HR max)	(85–90% HR max)
	R: 3′	R: 3′
	10′ cooling-down	10′ cooling-down
Session 3	8′ coordination warm-up with ball	8′ Coordination warm-up without ball
	Aerobic training 3 × 10′	Aerobic training 3 × 10′
	(85–90% HR max)	(85–90% HR max)
	R: 4′	R: 4′
	10′ cooling-down	10′ cooling-down

The active recovery period was conducted over two weeks with three sessions per week. Each session began with an 8 min coordination warm-up (alternating with or without the ball), followed by aerobic running intervals performed at 85–90% of maximum heart rate. Players completed either 3 × 10 min or 3 × 7 min runs, depending on the session, with 3–4 min of rest between intervals. Each session concluded with a 10 min cool-down.

**Table 6:** Effects of 8 weeks of LSCD vs. SSG training on anthropometric variables and body composition of study participants.

Variable	Group	Intervention	Pre	Post	Δ%	Within-Group Comparison (*p*, *d*)	Intervention Comparison (*p*, *d*)
Height (cm)	G1	LSCD	159.38 ± 8.46	159.38 ± 8.46	0.39 ± 5.31	*p* = 0.91. *d* = 0.01	*p* = 0.94. *d* = −0.02
		SSSG	159.77 ± 8.38	159.77 ± 8.38	0.38 ± 5.24	*p* = 0.91. *d* = 0.01	
	G2	LSCD	159.27 ± 4.66	159.44 ± 4.55	0.10 ± 2.89	*p* = 0.91. *d* = 0.01	*p* = 0.92. *d* = 0.06
		SSSG	159.14 ± 4.73	159.16 ± 4.73	0.01 ± 2.97	*p* = 0.95. *d* = 0.02	
Body mass (kg)	G1	LSCD	49.17 ± 9.73	50.02 ± 9.76	1.72 ± 19.82	*p* = 0.91. *d* = 0.04	*p* = 0.94. *d* = −0.10
		SSSG	50.48 ± 9.67	50.65 ± 9.59	0.32 ± 19.07	*p* = 0.91. *d* = 0.04	
	G2	LSCD	51.33 ± 6.18	50.86 ± 5.70	−0.92 ± 11.60	*p* = 0.91. *d* = 0.04	*p* = 0.96. *d* = −0.01
		SSSG	50.94 ± 6.49	51.41 ± 6.26	0.93 ± 12.52	*p* = 0.98. *d* = 0.01	
BMI (kg/m^2^)	G1	LSCD	22.17 ± 9.73	22.46 ± 9.76	1.30 ± 43.96	*p* = 0.91. *d* = 0.03	*p* = 0.94. *d* = −0.10
		SSSG	22.65 ± 9.67	22.75 ± 9.59	0.44 ± 42.52	*p* = 0.91. *d* = 0.03	
	G2	LSCD	23.39 ± 6.05	23.77 ± 6.03	1.59 ± 25.82	*p* = 0.91. *d* = 0.03	*p* = 0.96. *d* = −0.01
		SSSG	22.21 ± 5.60	22.54 ± 6.10	1.51 ± 26.40	*p* = 0.98. *d* = 0.01	
S6SF (cm)	G1	LSCD	80.86 ± 26.41	81.95 ± 26.60	1.35 ± 32.78	*p* = 0.80. *d* = −0.01	*p* = 0.84. *d* = −0.02
		SSSG	82.01 ± 26.89	81.96 ± 27.03	−0.06 ± 32.88	*p* = 0.80. *d* = −0.01	
	G2	LSCD	83.37 ± 23.64	84.13 ± 23.66	0.91 ± 28.37	*p* = 0.80. *d* = −0.01	*p* = 0.94. *d* = 0.06
		SSSG	82.24 ± 23.74	82.68 ± 23.73	0.53 ± 28.85	*p* = 0.92. *d* = 0.02	

**Table 7:** Effects of 8 weeks of LSCD vs. SSG training on fitness variables of study participants (mean ± SD).

Variable	Group	Intervention	Pre Mean ± SD	Post Mean ± SD	Δ% Mean ± SD	Within-Group Comparison (*p*, *d*)	Intervention Comparison (*p*, *d*)
5 m Sprint (s)	G1	LSCD	1.23 ± 0.05	1.13 ± 0.07	−8.43 ± 4.85	<0.001 (−2)	0.25 (−0.46)
		SSSG	1.28 ± 0.05	1.16 ± 0.06	−9.75 ± 4.33	<0.001 (−2.4)	
	G2	LSCD	1.36 ± 0.09	1.16 ± 0.09	−14.75 ± 6.63	<0.001 (−2.22)	0.38 (−0.35)
		SSSG	1.27 ± 0.08	1.19 ± 0.08	−6.75 ± 6.45	0.003 (−1)	
10 m Sprint (s)	G1	LSCD	2.16 ± 0.06	2.08 ± 0.06	−3.39 ± 2.78	<0.001 (−1.33)	0.002 (−1.33)
		SSSG	2.26 ± 0.06	2.12 ± 0.06	−6.46 ± 2.55	<0.001 (−2.33)	
	G2	LSCD	2.39 ± 0.10	2.16 ± 0.11	−9.64 ± 4.32	<0.001 (−2.3)	0.52 (−0.54)
		SSSG	2.24 ± 0.11	2.16 ± 0.11	−3.57 ± 5.01	0.04 (−0.73)	
20 m Sprint (s)	G1	LSCD	3.71 ± 0.11	3.67 ± 0.10	−1.13 ± 2.94	0.07 (−0.36)	0.11 (−0.48)
		SSSG	3.79 ± 0.29	3.57 ± 0.20	−5.86 ± 6.72	0.02 (−0.76)	
	G2	LSCD	3.79 ± 0.25	3.40 ± 0.29	−10.32 ± 7.12	<0.001 (−1.36)	0.56 (−0.23)
		SSSG	3.71 ± 0.30	3.33 ± 0.21	−10.31 ± 7.21	<0.001 (−1.35)	
*t*-test without ball (s)	G1	LSCD	9.85 ± 0.73	8.65 ± 0.32	−12.18 ± 6.42	<0.001 (−2.55)	0.29 (−0.42)
		SSSG	9.40 ± 0.61	8.49 ± 0.33	−9.69 ± 5.62	<0.001 (−2.13)	
	G2	LSCD	11.17 ± 0.65	8.38 ± 0.43	−24.97 ± 5.10	<0.001 (−4.2)	0.74 (−0.19)
		SSSG	10.98 ± 0.95	8.51 ± 0.28	−22.47 ± 7.71	<0.001 (−3.5)	
*t*-test with ball (s)	G1	LSCD	13.07 ± 0.49	11.25 ± 0.74	−13.93 ± 4.98	<0.001 (−2.42)	0.12 (−0.65)
		SSSG	13.56 ± 1.33	11.50 ± 0.96	−15.19 ± 8.77	<0.001 (−1.36)	
	G2	LSCD	12.88 ± 1.16	11.41 ± 1.34	−11.41 ± 9.76	0.001 (−1.08)	0.83 (−0.09)
		SSSG	13.17 ± 1.53	11.42 ± 1.01	−13.29 ± 10.21	0.001 (−1.19)	
SJ (cm)	G1	LSCD	22.66 ± 3.99	27.90 ± 4.06	23.12 ± 17.76	0.002 (1.32)	0.32 (0.45)
		SSSG	20.95 ± 2.74	26.46 ± 2.76	26.30 ± 13.12	<0.001 (1.97)	
	G2	LSCD	18.64 ± 2.52	25.26 ± 3.13	35.52 ± 15.41	<0.001 (2.54)	0.11 (−0.68)
		SSSG	21.71 ± 3.39	24.66 ± 3.89	13.59 ± 16.87	0.006 (0.86)	
CMJ with A (cm)	G1	LSCD	18.61 ± 3.29	23.56 ± 3.36	26.60 ± 17.87	<0.001 (1.51)	0.2 (−0.44)
		SSSG	19.28 ± 2.93	24.75 ± 3.82	28.37 ± 17.97	<0.001 (1.9)	
	G2	LSCD	18.81 ± 2.22	24.03 ± 2.51	27.75 ± 12.62	<0.001 (2.11)	0.04 (−0.97)
		SSSG	21.19 ± 2.97	25.87 ± 2.86	22.09 ± 13.75	<0.001 (1.61)	
CMJ without A (cm)	G1	LSCD	13.07 ± 0.49	19.21 ± 0.77	46.98 ± 5.14	<0.001 (8.25)	0.001 (1.73)
		SSSG	12.60 ± 0.84	17.58 ± 0.91	39.52 ± 6.96	<0.001 (6.55)	
	G2	LSCD	12.25 ± 1.43	19.27 ± 1.38	57.31 ± 11.47	<0.001 (5.01)	0.04 (−0.91)
		SSSG	14.17 ± 1.53	18.63 ± 1.05	31.47 ± 9.54	<0.001 (3.02)	

Data are presented as the mean and standard deviation. SJ: squat jump, CMJ with A: countermovement jump with arm, CMJ without A: countermovement jump without arm, LSCD: linear sprint and change of direction, SSSG: small-sided soccer games.

**Table 8:** Distribution of players according to menstrual cycle phase during the four physical tests (T1–T4) for the LSCD and SSSG groups (*n* = 27).

Groupe	Tests	EF	LF	ML	Total
LSCD and SSSG	T1	12 (44%)	5 (19%)	10 (37%)	27 (100%)
	T2	11 (41%)	6 (22%)	10 (37%)	27 (100%)
	T3	13 (48%)	4 (15%)	10 (37%)	27 (100%)
	T4	12 (44%)	5 (19%)	10 (37%)	27 (100%)

**Table 9:** sRPE during the mesocycle 1 periods (means ± standard deviations).

				95% Confidence Interval		
Mesocycle 1	Group	Average (Standard Deviation)	*p*-Value	Lower	Higher	*d*
Week 1	G1	1744.8 ± 130.5 AU	*p* = 0.95	−0.754	0.756	*d* = 0.001
	G2	1744.6 ± 127.7 AU				
Week 2	G1	1782.8 ± 160.4 AU	*p* = 0.93	−0.781	0.728	*d* = −0.02
	G2	1787.1 ± 162.9 AU				
Week 3	G1	1817.8 ± 53.4 AU	*p* = 0.56	−0.652	0.859	*d* = −0.05
	G2	1812.6 ± 44.2 AU				
Week 4	G1	1786.1 ± 130.5 AU	*p* = 0.94	−0.793	0.717	*d* = −0.03
	G2	1791.1 ± 131.7 AU				

**Table 10:** sRPE during the mesocycle 2 periods (averages ± standard deviations).

				95% Confidence Interval		
Mesocycle 2	Group	Average (Standard Deviation)	*p*-Value	Lower	Higher	*d*
Week 1	G1	1814 ± 58 AU	*p* = 0.16	−0.796	0.714	*d* = −0.04
	G2	1816.93 ± 79.5 AU				
Week 2	G1	1830.5 ± 106.7 AU	*p* = 0.70	−0.652	0.858	*d* = 0.10
	G2	1820.1 ± 93.2 AU				
Week 3	G1	1740.9 ± 32.7 AU	*p* = 0.53	−0.536	0.979	*d* = 0.22
	G2	1734 ± 27.7 AU				
Week 4	G1	1801.5 ± 78.7 AU	*p* = 0.57	−0.661	0.849	*d* = −0.09
	G2	1791.1 ± 131.7 AU				

## Data Availability

Data are available upon a reasonable request from corresponding authors.

## Abbreviations

LSCD	Linear sprints and changes of direction
SSSG	Small-sided soccer games
BMI	Body mass index
LSPT	Loughborough soccer passing test
RSSA	Repeat shuttle sprint ability test
Session-RPE	Session-perceived exertion rate
SJ	Squat jump
CMJ with A	Countermovement jump, with arms
CMJ without A	Countermovement jump, without arms
HRJ15	Height of repeated jumps
PRJ15	Power of repeated jumps
*t*-Test	*t*-Test without ball and without ball
YIRT 1	Yo-Yo Intermittent Recovery Test Level 1
